# Establishment of *Anthoceros agrestis* as a model species for studying the biology of hornworts

**DOI:** 10.1186/s12870-015-0481-x

**Published:** 2015-04-09

**Authors:** Péter Szövényi, Eftychios Frangedakis, Mariana Ricca, Dietmar Quandt, Susann Wicke, Jane A Langdale

**Affiliations:** Institute of Evolutionary Biology and Environmental Studies, University of Zurich, Zurich, Switzerland; Institute of Systematic Botany, University of Zurich, Zurich, Switzerland; Swiss Institute of Bioinformatics, Quartier Sorge-Batiment Genopode, Lausanne, Switzerland; MTA-ELTE-MTM Ecology Research Group, ELTE, Biological Institute, Budapest, Hungary; Department of Plant Sciences, University of Oxford, South Parks Rd, Oxford, UK; Nees-Institut für Biodiversität der Pflanzen, University of Bonn, Meckenheimer Allee 170, D – 53115 Bonn, Germany; Institute for Evolution and Biodiversity, University of Muenster, Huefferstr. 1, 48149 Muenster, Germany; Current Address: Graduate School of Science, University of Tokyo, 7-3-1 Hongo, Bunkyo-ku, Tokyo 113 0033 Japan

**Keywords:** Bryophytes, Non-seed plants, Model species, Development, Evolution, Sporophyte, Genetically divergent strains

## Abstract

**Background:**

Plants colonized terrestrial environments approximately 480 million years ago and have contributed significantly to the diversification of life on Earth. Phylogenetic analyses position a subset of charophyte algae as the sister group to land plants, and distinguish two land plant groups that diverged around 450 million years ago – the bryophytes and the vascular plants. Relationships between liverworts, mosses hornworts and vascular plants have proven difficult to resolve, and as such it is not clear which bryophyte lineage is the sister group to all other land plants and which is the sister to vascular plants. The lack of comparative molecular studies in representatives of all three lineages exacerbates this uncertainty. Such comparisons can be made between mosses and liverworts because representative model organisms are well established in these two bryophyte lineages. To date, however, a model hornwort species has not been available.

**Results:**

Here we report the establishment of *Anthoceros agrestis* as a model hornwort species for laboratory experiments. Axenic culture conditions for maintenance and vegetative propagation have been determined, and treatments for the induction of sexual reproduction and sporophyte development have been established. In addition, protocols have been developed for the extraction of DNA and RNA that is of a quality suitable for molecular analyses. Analysis of haploid-derived genome sequence data of two *A. agrestis* isolates revealed single nucleotide polymorphisms at multiple loci, and thus these two strains are suitable starting material for classical genetic and mapping experiments.

**Conclusions:**

Methods and resources have been developed to enable *A. agrestis* to be used as a model species for developmental, molecular, genomic, and genetic studies. This advance provides an unprecedented opportunity to investigate the biology of hornworts.

## Background

Plants colonized terrestrial environments approximately 480 million years ago [1,2]. Phylogenetic analyses position one or more groups of charophyte algae as the sister group to land plants and reveal two distinct groups of land plants: the bryophytes and the monophyletic group of vascular plants [3]. The bryophytes comprise three monophyletic lineages, the liverworts, the mosses and the hornworts. Although subject to much scrutiny, the phylogenetic relationship between these three lineages remains fiercely debated [3-9]. The widely accepted view, supported by phylogenomic analyses [3], is that liverworts, mosses and hornworts branch as successive sister groups such that hornworts are the sister to vascular plants. However, more recent analyses based on protein sequences suggested that the position of hornworts as vascular plant sister group is an artefact of convergent codon usage in the two lineages [8]. Moreover, the data supported monophyly of liverworts and mosses, a relationship that is further validated by phylotranscriptomic analyses of a much larger taxon group [9]. Depending on the phylogenetic method used, this latest study identified hornworts as either sister to all land plants, in a clade with mosses and liverworts, or sister to vascular plants [9].

The uncertainty over the phylogenetic position of hornworts is compounded by our relatively limited understanding of hornwort biology. As land plants evolved, the modification of various character traits led to a general increase in size and complexity such that the bryophytes are relatively simple, both in terms of morphology and physiology, as compared to flowering plants. An understanding of how this complexity evolved can be obtained through comparative analyses of developmental processes in extant land plant species. To date, the liverwort *Marchantia polymorpha* and the moss *Physcomitrella patens* have been used to reveal evolutionary trajectories of developmental mechanisms that regulate morphological traits such as root hairs [10], and both endogenous (e.g. hormone signaling [11]) and environmentally-induced (e.g. chloroplast function [12]) physiological traits. However, such analyses have not been possible in hornworts because no species has thus far proved amenable to experimental manipulation in the laboratory.

Regardless of whether hornworts are sister to all other land plants, sister to vascular plants, or part of a bryophyte clade, their phylogenetic position is key to understanding the evolution of land plant body plans [13-15]. Notably, hornworts exhibit a number of morphological features that are distinct from those in liverworts and mosses, and thus they represent the only bryophyte lineage that can be effectively utilized for comparative analyses [16]. For example, the first zygotic division in hornworts is longitudinal whereas it is transverse in liverworts and mosses [17,18]; hornworts are the only land plants to develop chloroplasts with algal-like pyrenoids [19,20]; and hornworts characteristically have a symbiotic relationship with *Nostoc* cyanobacteria [16]. An understanding of how these biological processes are regulated and have evolved can only be achieved using a hornwort model system that can be easily grown throughout the entire life-cycle in laboratory conditions.

**Figure 1:**
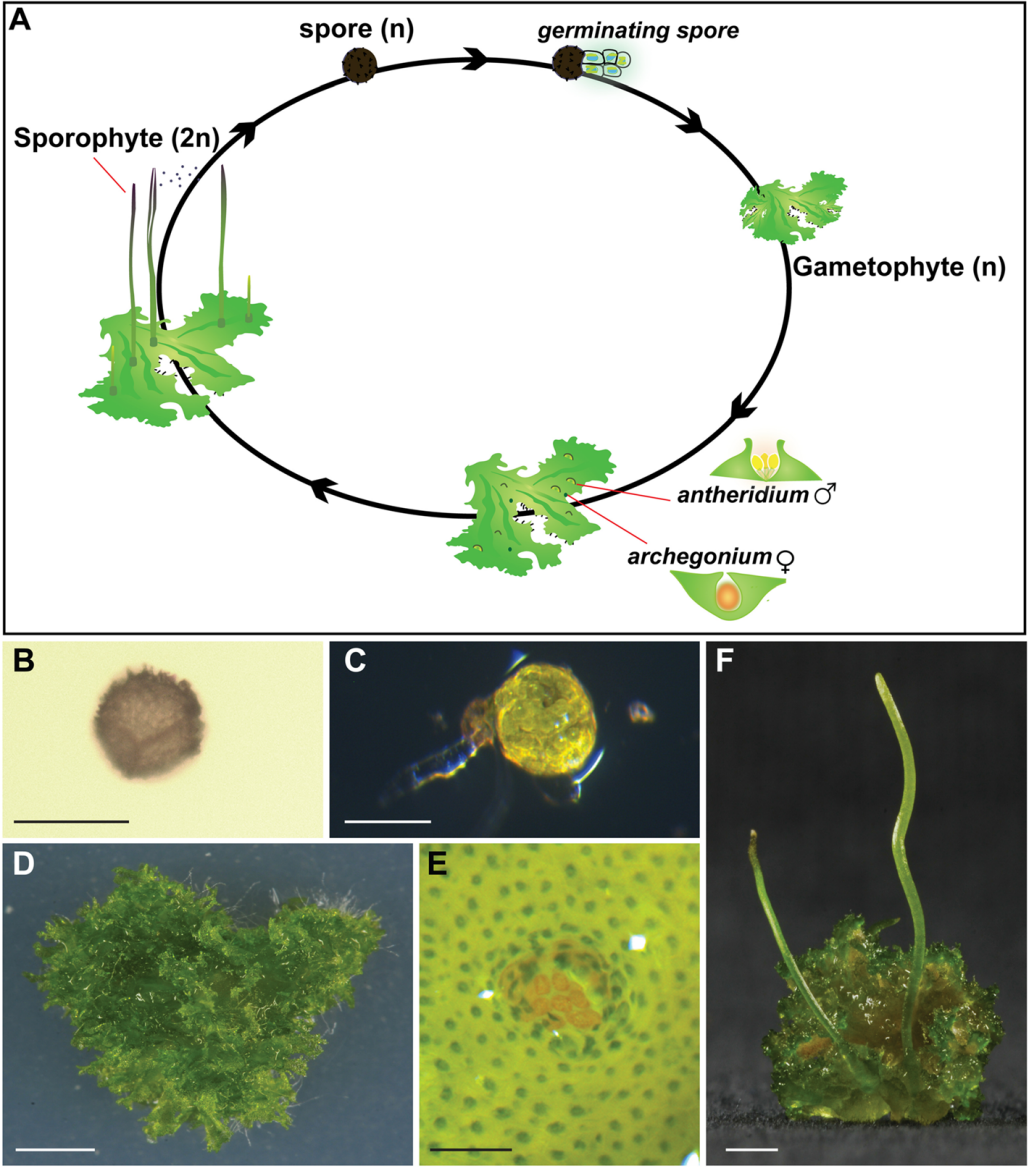
**Life cycle of the hornwort** ***Anthoceros agrestis*** **.** The life cycle of *A. agrestis* **(A)** starts with the spore **(B)** that germinates **(C)** and gives rise to the gametophyte **(D)**. Gametophytes are monoecious and thus individual plants bear both male antheridia **(E)** and female archegonia. After fertilization of the egg by sperm from the antheridia, the zygote is retained within the archegonium. The resultant embryo develops into the sporophyte **(F)** in which spores are produced via meiosis. Scale bars = B: 40 μm; C: 100 μm; D: 2 mm; E: 200 μm; F: 2 mm.

Here we introduce *Anthoceros agrestis* as a tractable hornwort experimental system. *Anthoceros* was the first hornwort genus described [21], it has worldwide distribution [22], most species have small genomes [23] with *A. agrestis* having the smallest genome of all bryophytes investigated so far (1C = 0.085 pg ca. 83 Mbp [Megabase pairs]; [24]). Similar to all bryophytes, the haploid gametophyte generation of *A. agrestis* is the dominant phase of the life cycle (Figure 1A). Spores germinate to produce a flattened thallus that generally lacks specialized internal tissue differentiation with the exception of cavities that contain mucilage (Figure 1B-D, [16]). Each cell of the thallus (including the epidermal cells) contains one to four chloroplasts [16]. Gametophytes are monoecious with both male (antheridia) and female (archegonia) reproductive organs developing on the same thallus. Antheridia develop in chambers (up to 45 per chamber) (Figure 1E, [16]) and produce motile sperm, whereas archegonia contain a single egg that is retained in the thallus. After fertilization, the diploid embryo develops within the archegonium to produce the sporophyte, in which spores are produced via meiosis. At maturity the *A. agrestis* sporophyte is an elongated cylindrical structure (Figure 1F) that is composed of the columella, a spore layer, a multicellular jacket and elaters for spore dispersal [16]. The meristem at the base of the sporophyte (basal meristem) remains active throughout the life of the sporophyte, a feature that is unique to hornworts [16]. The propagation of *A. agrestis* callus and suspension cultures has previously been reported for biochemical analyses [25]. Here, we report the development of methods and resources to grow and propagate *A. agrestis* axenically, to facilitate molecular analysis, and to generate populations for genetic analysis

## Results and discussion

### *A. agrestis* strains

Two different *A. agrestis* strains have been propagated. The first was established from plant material collected near Fogo in Berwickshire, UK (hereafter referred to as the “Oxford strain”) and the second from plant material collected near Hirschbach, Germany (hereafter referred to as the “Bonn strain”). All existing material of both the Oxford and Bonn strains originate from a single spore. Attempts to establish *Anthoceros punctatus* strains were carried out in parallel, and although vegetative propagation was successful, conditions for reproductive propagation proved elusive. As such, *A. punctatus* was rejected as a potential model organism.

### Establishment of axenic cultures

To initiate axenic cultures, several sterilization protocols were tested. Bacterial and fungal contamination of spores was successfully eliminated using bleach, and thus a simple three-minute treatment followed by washing was adopted (see Methods). Following sterilization, spores were germinated on Lorbeer’s medium, a substrate that has previously been used for hornwort cultivation [26]. Germination occurred after approximately 7 days when plates were incubated at 23°C, with a diurnal cycle of 16 h light (300 μEm^2^sec^−1^)/8 h dark. Young gametophytes were large enough to be sub-cultured 1–2 months after spore germination.

### Gametophyte cultures and vegetative propagation

Three different media were tested for their ability to support vegetative growth of gametophytes. In addition to Lorbeer’s medium, gametophytes were transferred to 1/10 KNOP medium [27] and to BCD [28] medium, both of which have been previously used to culture the moss *P. patens*. Plates were incubated at 23°C, either under a diurnal cycle of 16 h light (300 μEm^2^sec^−1^)/8 h dark or under continuous light (300 μEm^2^sec^−1^). In all cases, cultures were propagated and maintained by monthly sub-culturing, in which a small fragment of thallus tissue (~5-7 mm in diameter) was cut and placed on fresh medium. In general, the Oxford strain grew better (faster, greener and healthier) on Lorbeer’s or 1/10 KNOP media whereas the Bonn strain grew better on BCD medium. In all cases, plants grew faster under continuous light than with long day photoperiods, as long as the light intensity was kept at, or below, 300 μEm^2^sec^−1^.

### Sporophyte induction and sexual reproduction

In natural ecosystems, sexual reproduction in hornworts is initiated by the formation of antheridia on the thallus, and then after approximately one month archegonia develop [29]. To determine the conditions under which this developmental transition towards gametangia formation can be induced in the laboratory, growth parameters were varied. Cultures were initiated by either sub-culturing thallus fragments (as above) or by germinating spores, with thallus fragments being preferable starting material because the time from spore germination to the development of thallus that was mature enough for reproductive induction was around 2–3 months. The most significant factor that influenced whether gametophytes grew vegetatively or formed gametangia was growth temperature. Effective induction of gametangia was achieved by dropping the growth temperature of gametophyte cultures from 23°C to 16°C.

To optimize induction conditions, growth at 16°C was next compared on different media and under different light regimes. Gametangia were successfully induced on both 1/10 KNOP and BCD media but not on Lorbeer’s medium, and in both continuous light (150 μEm^2^sec^−1^) or long day photoperiod 16 h light (150 μEm^2^sec^−^1)/8 h dark. In all cases, antheridia appeared as reddish dots on the surface of the thallus after approximately one month. Given that archegonia are colourless and are embedded within the thallus, their formation could not be easily visualized, and thus the appearance of antheridia was used as a prompt to induce fertilization.

Fertilization was facilitated by adding 5–10 mL of either water or liquid culture media to each culture. Sporophytes were visible after another month of growth. However, the number of sporophytes produced per thallus was increased if the liquid addition step was repeated 3–5 times over a period of ~2 weeks after addition of the first aliquot. Presumably the increased number of successful fertilization events results from variation in the timing of archegonium formation (i.e. it is likely that when the first aliquot was added very few archegonia were present). This variability is also reflected in the fact that even with the extra liquid addition steps, the number of sporophytes produced by each thallus ranged from 5 to over 100. There is no apparent way in which this variation can be more carefully controlled given that the development of archegonia is difficult to monitor. Emerging sporophytes went through the normal cycle of sporophyte maturation and contained hundreds of spores. Spores were viable and were regularly used to initiate new cultures.

### Nucleic acid extraction

Although the extraction of nucleic acids from any organism is generally considered to be straightforward, hornwort gametophyte tissue is rich in polysaccharides (mucilage) [16], and was also found to be rich in polyphenolics. Both compounds pose a problem for DNA and RNA extraction. A range of DNA and RNA extraction protocols were therefore tested to optimize the procedure and to reduce contamination levels as much as possible. A modified CTAB protocol, adapted from Porebski et al. [30], was found to be optimal for genomic DNA extraction in that yields were approximately ten times higher than standard CTAB protocols. This protocol uses polyvinylpyrrolidone to remove polyphenolics and contains an extra ethanol precipitation step with a relatively high NaCl concentration compared to standard DNA extraction protocols. At NaCl concentrations higher than 0.5 M, polysaccharides remain in solution and do not co-precipitate with DNA. The overall yield of DNA extracted was also highly dependent on the conditions under which the thalli were grown. Thalli grown on petri dishes in which extra liquid medium (~5-10 mL per 9 cm diameter petri dish) was added every 2–3 weeks to maintain a liquid film (1–2 mm thick) connecting the thalli on the surface of the agar yielded the greatest amounts of DNA. In addition, less, rather than more plant material led to the highest yields. Optimal yields were obtained in extractions that used 1–2 thalli, each of ~0.5 cm in diameter, that had been grown under wet conditions. DNA extracted with this protocol was successfully used in next-generation sequencing library preparation, for restriction enzyme digests, and in PCR reactions. The same protocol could be used for RNA extraction with the addition of an overnight RNA precipitation step with LiCl (see Methods).

### Genome-wide genetic divergence of the Oxford and Bonn strains of *A. agrestis*

The haploid genome size of *A. agrestis* has previously been reported as 83Mbp on the basis of flow cytometry [24]. Using k-mer analysis we estimated the Bonn strain to have a haploid genome size of approximately 71Mbp (70981934 bp), a number consistent with that derived from flow cytometry. The genome size was further confirmed by the total length of the draft assembly (approximately 90 Mbp, Bonn strain). To determine the extent to which the Bonn and Oxford strains are different at the nucleotide level, we resequenced the Oxford strain and mapped the reads onto the Bonn assembly. On average we found approximately 2 single nucleotide polymorphisms (SNPs) per 1 Kbp (Kilobase pairs) sequence data (1.996 SNPs). This is less than that reported for accessions of *Arabidopsis thaliana* (5 SNPs/1 Kbp) [31] or *Populus tremula* (2–6 SNPs/1 Kbp) [32], but is of the same order of magnitude. This level of variation is likely to be sufficient to conduct classical genetic work and gene mapping by sequencing, as reported for the moss *P. patens* where strains show a similar level of genetic divergence [33].

## Conclusions

Methods and resources have been developed to enable *A. agrestis* to be used as a model species for developmental, molecular, genomic and genetic studies. Axenic cultures have been established, conditions for sexual propagation and nucleic acid extraction have been optimised, and two strains with sufficient genetic divergence have been identified for genetic analyses. This advance provides an unprecedented opportunity to investigate the biology of hornworts.

### Availability of supporting data

Raw sequence data for the Bonn and Oxford strains have been deposited in the European Nucleotide Archive and are available under study accession number PRJEB8683 (http://www.ebi.ac.uk/ena/data/view/PRJEB8683).

## Methods

### Plant material

The *Anthoceros agrestis* Oxford strain was obtained from Berwickshire (Berwickshire, near Fogo, Grid: NT 7700 4894, v.-c. 81, Alt. c. 115 m) on 30^th^ October 2012 by Dr David Long (Royal Botanic Garden Edinburgh). Voucher specimens have been deposited in the Fielding Druce Herbarium, University of Oxford (OXF). The *A. agrestis* Bonn strain was obtained between Hirschbach and Reinhardtsgrimma, on a crop field approximately 500 m from the street (K9022), near a small copse on 15^th^ November 2006 by Dr Susann Wicke and Dr Dietmar Quandt. Voucher specimens have been deposited in the Herbarium of the University of Bonn (H015-H018).

### Growth media

Three different media were used: Lorbeer’s medium [26] (0.1 g/L MgSO_4_.7H_2_O, 0.1 g/L KH_2_PO_4_, 0.2 g/L NH_4_NO_3_, 0.1 g/L CaCl_2_) supplemented with 1 mL of Hutner’s trace elements [34] (50 g/L EDTA disodium salt, 22 g/L ZnSO_4_.7H_2_O, 11.4 g/L H_3_BO_3_, 5.06 g/L MnCl_2_.4H_2_O, 1.61 g/L CoCl_2_.6H_2_O, 1.57 g/L CuSO_4_.5H_2_O, 1.1 g/L (NH_4_)_6_Mo_7_O_24_.4 H_2_O, 4.99 g/L FeSO_4_.7H_2_O) adjusted to pH6.5 and solidified with 6.5 g/L agar; 1/10 KNOP medium [27] (0.025 g/L K_2_HPO_4,_ 0.025 g/L KH_2_PO_4_, 0.025 g/L KCl, 0.025 g/L MgSO_4_.7H_2_O, 0.1 g/L Ca(NO_3_)_2_.4H_2_O, 37 mg/L FeSO_4_.7H_2_O) adjusted to pH6.5 and solidified with 6.5 g/L agar; and BCD medium [28] (0.25 g/L MgSO_4_.7H_2_O, 0.25 g/L KH_2_PO_4_ (pH6.5), 1.01 g/L KNO_3_, 0.0125 g/L FeSO_4_.7H_2_O and 0.001% Trace Element Solution (0.614 mg/L H_3_BO_3_, 0.055 mg/L AlK(SO_4_)2.12H_2_O, 0.055 mg/L CuSO_4_.5H_2_O, 0.028 mg/L KBr, 0.028 mg/L LiCl, 0.389 mg/L MnCl_2_.4H_2_O, 0.055 mg/L CoCl_2_.6H_2_O, 0.055 mg/L ZnSO_4_.7H_2_O, 0.028 mg/L KI and 0.028 mg/L SnCl_2_.2H_2_O) supplemented with 1mM CaCl_2_ and solidified with 8 g/L agar.

### Tissue sterilization

Isolated sporophytes were left to dry before removing the spore contents. Spores were sterilized by gentle agitation in 5% (v/v) bleach (sodium hypochlorite solution, ~10%) in microcentrifuge tubes, followed by three washes in sterile water with brief centrifugation steps between each wash.

### Genomic DNA extraction

DNA was extracted from 1 gram of ground frozen tissue using 10 mL prewarmed (60°C) extraction buffer (100 mM Tris–HCl pH8, 1.4M NaCl, 20 mM EDTA pH8, 2% (w/v) CTAB, 0.3% (v/v) β-mercaptoethanol, 100 mg of polyvinylpyrrolidone-40 (PVP) per 1 g tissue) plus 5 μl of 100 mg/mL RNAase A. After incubation at 60°C for 30 min, samples were cooled to room temperature and then extracted with chloroform:isoamylalcohol (24:1). A second chloroform:isoamylalcohol (24:1) step was carried out to remove any remaining PVP. DNA was precipitated from the aqueous phase with 0.5 volumes 5M NaCl and 2 volumes of cold (−20°C) 95% ethanol. After resuspension in 2 mL 10 mM Tris pH8, 1mM EDTA (TE), a second ethanol precipitation was carried out and then the DNA was dissolved in TE for storage and subsequent analyses.

### RNA extraction

RNA was extracted in two different ways. For large scale RNA extractions, samples were treated as for DNA extractions with the exception that all solutions were prepared with water that had been autoclaved after treatment with 0.1% diethylpyrocarbonate (DEPC) and RNAase was omitted from the extraction buffer. In addition, after the second ethanol precipitation, the pellet was resuspended in DEPC-treated dH_2_O instead of TE. RNA was then precipitated overnight at 4°C after the addition of 0.25 volumes of 8 M LiCl. After resuspension and a third ethanol precipitation, RNA was resuspended in DEPC-treated water for storage at −80°C and subsequent analyses.

For extractions where the recovery of small RNAs was required, the Spectrum™ Plant Total RNA Kit (Sigma) was used. Before each extraction residual water was removed from ~2-3 thalli (each ~0.5 cm diameter) using paper towel. Tissue was flash-frozen in liquid N_2_, ground into a fine powder and resuspended in 750 μL binding buffer. RNA was eluted in 30 + 30 μL of nuclease free water and stored at −80°C.

### Sequence analysis

To generate a low-coverage reference sequence for the *A. agrestis* Bonn strain, DNA was extracted from one month old thalli using the protocol detailed above. The draft genome sequence data are derived from the haploid phase, a significant advantage over vascular-plant genomes, which are all based on diploid individuals. Paired-end libraries were prepared for next generation sequencing using the Nextera XT kit (Illumina inc.) with 1 to 10 ng DNA. Nextera DNA libraries were sequenced on 1/3^rd^ of a Miseq flow cell with 250 cycles. After sequencing and de-multiplexing, approximately 4.99 million paired-end reads were obtained. Reads were trimmed using Trimmomatic [35] and all reads that were 36 bp or longer after quality trimming and filtering (−phred33 ILLUMINACLIP: NexteraPE-PE.fa:2:30:10:8:true LEADING:9 TRAILING:3 SLIDINGWINDOW:4:15 MINLEN:36) were retained. The resultant 4.94 million paired-end reads were assembled using the udba500 code (part of the A5 pipeline; [36]) with k-mer values ranging from 20 to 230 and a step size of 20. To verify the validity of previous estimates of genome size [24] k-mer analysis was used as implemented in the code kmergenie (version 1.6950; [37]). To identify SNPs between the Bonn and the Oxford strains, the Oxford strain was resequenced as above. We obtained approximately 2.33 million raw paired-end reads of which 2.29 million reads survived quality filtering and trimming as described above. This sequencing depth corresponds to a theoretical average coverage of 8x. Raw sequence data of the Bonn and Oxford strains will be deposited in the SRA archive upon acceptance of the manuscript for publication.

### SNP discovery

GATK (Genome analysis toolkit) best practice was followed to identify SNPs with high-confidence [38]. Briefly, we mapped cleaned and trimmed reads to the Bonn strain`s preliminary assembly using bowtie2 (bowtie2_2.1.0, using the --sensitive option; [39]). Duplicates were then marked and removed using the picard tool MarkDuplicates module (http://broadinstitute.github.io/picard/) and reads re-aligned using the GATK IndelRealigner [40]. Finally, we used SNVer [41] to extract SNPs between the Bonn and the Oxford strains (−n 1 -mq 20 -bq 17 -b 0.75 -het 0.0001 -a 1 -s 0.0001). Because the Oxford strain was resequenced with low coverage, SNPs were called at all positions with a coverage value greater than five. Finally, we used vcftools [42] to calculate the density of SNPs in 1 Kbp windows. For this analysis we excluded all contigs from the Bonn strain assembly that were shorter than 1 Kbp.

## Abbreviations

CTAB: Cetyltrimethylammonium bromide
GATK: Genome analysis toolkit
Mbp: Megabase pairs
Kbp: Kilobase pairs
SNPs: Single nucleotide polymorphisms
PVP: Polyvinylpyrrolidone
TE: Tris EDTA
EDTA: Ethylenediaminetetraacetic acid
DEPC: Diethylpyrocarbonate

## References

[1] Gensel PG (2008). The earliest land plants. Ann Rev Ecol Evol.

[2] Kenrick P, Crane PR (1997). The origin and early evolution of plants on land. Nature.

[3] Qiu YL, Li L, Wang B, Chen Z, Knoop V, Groth-Malonek M (2006). The deepest divergences in land plants inferred from phylogenomic evidence. Proc Natl Acad Sci.

[4] Chang Y, Graham SW (2011). Inferring the higher-order phylogeny of mosses (Bryophyta) and relatives using a large, multigene plastid data set. Am J Bot.

[5] Nickrent DL, Parkinson CL, Palmer JD, Duff RJ (2000). Multigene phylogeny of land plants with special reference to bryophytes and the earliest land plants. Mol Biol Evol.

[6] Nishiyama T, Wolf PG, Kugita M, Sinclair RB, Sugita M, Sugiura C (2004). Chloroplast phylogeny indicates that bryophytes are monophyletic. Mol Biol Evol.

[7] Qiu YL, Cho Y, Cox JC, Palmer JD (1998). The gain of three mitochondrial introns identifies liverworts as the earliest land plants. Nature.

[8] Cox CJ, Li B, Foster PG, Embley TM, Civan P (2014). Conflicting phylogenies for early land plants are caused by composition biases among synonymous substitutions. Syst Biol.

[9] Wickett NJ, Mirarab S, Nguyen N, Warnow T, Carpenter E, Matasci N (2014). Phylotranscriptomic analysis of the origin and early diversification of land plants. Proc Natl Acad Sci.

[10] Menand B, Yi K, Jouannic S, Hoffmann L, Ryan E, Linstead P (2007). An ancient mechanism controls the development of cells with a rooting function in land plants. Science.

[11] Yasumura Y, Crumpton-Taylor M, Fuentes S, Harberd NP (2007). Step-by-step acquisition of the gibberellin-DELLA growth-regulatory mechanism during land-plant evolution. Curr Biol.

[12] Yasumura Y, Moylan E, Langdale J (2005). A conserved transcription factor mediates nuclear control of organelle biogenesis in anciently diverged land plants. Plant Cell.

[13] Ligrone R, Duckett JG, Renzaglia KS (2012). Major transitions in the evolution of early land plants: a bryological perspective. Ann Bot.

[14] Tomescu AM, Wyatt SE, Hasebe M, Rothwell GW (2014). Early evolution of the vascular plant body plan - the missing mechanisms. Curr Opin Plant Biol.

[15] Rothwell GW, Wyatt SE, Tomescu AM (2014). Plant evolution at the interface of paleontology and developmental biology: An organism-centered paradigm. Am J Bot.

[16] Renzaglia KS, Villarreal JC, Duff RJ (2009). New Insights into Morphology, Anatomy and Systematics of Hornworts. Bryophyte Biology II.

[17] Renzaglia KS (1978). A comparative morphology and developmental anatomy of the anthocerotophyta. J Hattori Bot Lab.

[18] Ligrone R, Duckett JG, Renzaglia KS (2012). The origin of the sporophyte shoot in land plants: a bryological perspective. Ann Bot.

[19] Villarreal JC, Renner SS (2012). Hornwort pyrenoids, carbon-concentrating structures, evolved and were lost at least five times during the last 100 million years. Proc Natl Acad Sci.

[20] Duckett JG, Renzaglia KS (1988). Ultrstructure and development of plastids in bryophytes. Adv Bryology.

[21] Merrett C. Pinax rerum naturalium Britannicarum: continens vegetabilia, animalia, et fossilia, in hac insula reperta inchoatus, London; 1667.

[22] Villarreal JC, Cargill DC, Hagborg A, Soderstrom L, Renzaglia KS (2010). A synthesis of hornwort diversity: patterns, causes and future work. Phytotaxa.

[23] Bainard JD, Villarreal JC (2013). Genome size increases in recently diverged hornwort clades. Genome.

[24] Leitch IJ, Bennett MD (2007). Genome Size and Its Uses: The Impact of Flow Cytometry. Flow Cytometry with Plant Cells: Analysis of Genes Chromosomes and Genomes.

[25] Vogelsang K, Schneider B, Petersen M (2006). Production of rosmarinic acid and a new rosmarinic acid 3’-O-ß-D-glucoside in suspension cultures of the hornwort *Anthoceros agrestis* Paton. Planta.

[26] Proskauer JM (1969). Studies on Anthocerotales. VIII Phytomorphology.

[27] Reski R, Abel WO (1985). Induction of budding on chloronemata and caulonemata of the moss, *Physcomitrella patens*, using isopentenyladenine. Planta.

[28] Cove DJ, Perroud PF, Charron AJ, McDaniel SF, Khandelwal A, Quatrano RS. Culturing the moss Physcomitrella patens. Cold Spring Harb Protoc 2009, 2009:pdb prot5136. 10.1101/pdb.prot5136 20147066

[29] Proskauer JM (1967). Studies on Anthocerotales VII. Phytomorphology.

[30] Porebski S, Bailey LG, Baum B (1997). Modification of a CTAB DNA extraction protocol for plants containing high polysaccharide and polyphenol components. Plant Mol Biol Rep.

[31] Gan X, Stegle O, Behr J, Steffen JG, Drewe P, Hildebrand KL (2011). Multiple reference genomes and transcriptomes for *Arabidopsis thaliana*. Nature.

[32] Zhou L, Bawa R, Holliday JA (2014). Exome resequencing reveals signatures of demographic and adaptive processes across the genome and range of black cottonwood (*Populus trichocarpa*). Mol Ecol.

[33] Kamisugi Y, von Stackelberg M, Lang D, Care M, Reski R, Rensing SA (2008). A sequence-anchored genetic linkage map for the moss, *Physcomitrella patens*. Plant J.

[34] Hutner SH, Provasoli L, Schatz A, Haskins CP (1950). Some approaches to the study of the role of metals in the metabolism of microrganisms. Proc Am Phil Soc.

[35] Bolger AM, Lohse M, Usadel B (2014). Trimmomatic: a flexible trimmer for Illumina sequence data. Bioinformatics.

[36] Coil D, Jospin G, Darling AE. A5-miseq: an updated pipeline to assemble microbial genomes from Illumina MiSeq data. Bioinformatics 2014, doi:10.1093/bioinformatics/btu661. 10.1093/bioinformatics/btu661 25338718

[37] Chikhi R, Medvedev P (2014). Informed and automated *k*-mer size selection for genome assembly. Bioinformatics.

[38] McKenna A, Hanna M, Banks E, Sivachenko A, Cibulskis K, Kernytsky A (2010). The Genome Analysis Toolkit: a MapReduce framework for analyzing next-generation DNA sequencing data. Genome Res.

[39] Langmead B, Salzberg S (2012). Fast gapped-read alignment with Bowtie 2. Nat Methods.

[40] DePristo M, Banks E, Poplin R, Garimella K, Maguire J, Hartl C (2011). A framework for variation discovery and genotyping using next-generation DNA sequencing data. Nat Genet.

[41] Wei Z, Wang W, Hu P, Lyon GJ, Hakonarson H (2011). SNVer: a statistical tool for variant calling in analysis of pooled or individual next-generation sequencing data. Nucl Acids Res.

[42] Danecek P, Auton A, Abecasis G, Albers CA, Banks A, DePristo MA (2011). The variant call format and VCFtools. Bioinformatics.

